# Effect of mountain ultramarathon distance competition on biochemical variables, respiratory and lower-limb fatigue

**DOI:** 10.1371/journal.pone.0238846

**Published:** 2020-09-11

**Authors:** Ignacio Martínez-Navarro, Juan Miguel Sanchez-Gómez, Inma Aparicio, Jose Ignacio Priego-Quesada, Pedro Pérez-Soriano, Eladio Collado, Bárbara Hernando, Carlos Hernando

**Affiliations:** 1 Physical Education and Sports Department, University of Valencia, Valencia, Spain; 2 Sports Health Unit, Vithas 9 de Octubre Hospital, Valencia, Spain; 3 University Clinical Hospital of Valencia, Valencia, Spain; 4 Research Group in Sports Biomechanics (GIBD), Physical Education and Sports Department, University of Valencia, Valencia, Spain; 5 AITEX (Textil Research Institute), Alcoy, Spain; 6 Faculty of Health Sciences, Jaume I University, Castellon, Spain; 7 Department of Medicine, Jaume I University, Castellon, Spain; 8 Sport Service, Jaume I University, Castellon, Spain; 9 Department of Education and Specific Didactics, Jaume I University, Castellon, Spain; Universidade Federal de Mato Grosso do Sul, BRAZIL

## Abstract

The study aimed at assessing the acute physiological effects of running a 65-km vs a 107-km mountain ultramarathon. Nineteen athletes (15 males and 4 females) from the shorter race and forty three athletes (26 males and 17 females) from the longer race were enrolled. Body weight, respiratory and lower limb strength were assessed before and after the race. Blood samples were obtained before, after and 24-h post-race. Body weight loss did not differ between races. A decrease in squat jump height (p<0.01; d = 1.4), forced vital capacity (p<0.01; d = 0.5), forced expiratory volume in 1 s (p<0.01; d = 0.6), peak inspiratory flow (p<0.01; d = 0.6) and maximal inspiratory pressure (p<0.01; d = 0.8) was observed after the longer race; while, after the shorter race only maximal inspiratory pressure declined (p<0.01; d = 0.5). Greater post-race concentrations of creatine kinase (p<0.01; d = 0.9) and C-reactive protein (p<0.01; d = 2.3) were observed following the longer race, while high-sensitivity cardiac troponin was higher after the shorter race (p<0.01; d = 0.3). Sodium decreased post-competition only after the shorter race (p = 0.02; d = 0.6), while creatinine increased only following the longer race (p<0.01; d = 1.5). In both groups, glomerular filtration rate declined at post-race (longer race: p<0.01, d = 2.1; shorter race: p = 0.01, d = 1.4) and returned to baseline values at 24 h post-race. In summary, expiratory and lower-limb fatigue, and muscle damage and inflammatory response were greater following the longer race; while a higher release of cardiac troponins was observed after the shorter race. The alteration and restoration of renal function was similar after either race.

## 1. Introduction

The popularity of mountain ultramarathons (MUM) has grown exponentially during the last few years [1, 2] and they constitute an outstanding model for the study of the acute consequences of ultra-endurance exercise on human body physiology [3]. Indeed, several investigations have documented the effects of running a MUM on lower-limb neuromuscular fatigue [4–7], respiratory fatigue [8, 9], cardiac damage [10–13], renal function and inflammatory activity [4, 14–18]. These data is important for both medical and coaching communities, to increase the knowledge of potential side effects and a harmful influence on health of running a MUM [19], on one hand; and, on the other hand, to help coaches to better plan their athletes’ training sessions following the race [20]. However, a considerably lower number of studies have compared how MUM distance may affect the abovementioned responses [5, 21].

It has been suggested that extremely long distance MUM (i.e., 330-km) provoke lesser neuromuscular fatigue, muscle damage and inflammation than shorter ones (i.e., 110–166 km) [5]. This might be explained because runners adopt a more conservative pacing strategy according to the distance to avoid early abandonment and preserve muscle integrity [22, 23]. When comparing mountain races of ~50 km and ~100 km, it appears that longer races induce greater inflammatory and muscle damage responses while cardiac damage biomarkers release is lower [21]. On the other hand, significant reductions in expiratory pulmonary function have been reported following both a 110-km [9] and a 330-km MUM [8]. Nevertheless, most of those studies have focused their analyses on a specific physiological response; so further studies that integrate biochemical data, lower-limb and respiratory fatigue variables are needed to improve the interpretation of the results. Having a broader picture of how ultramarathon distance competition affects physiology and performance could ease the understanding of athletes’ in-race performance and post-race recovery [22].

The aim of our study was, therefore, to assess the acute physiological effects of running a 65-km vs a 107-km MUM from an integrative perspective. Specifically, we were interested in analyzing and comparing body weight loss and sodium depletion, lower-limb and respiratory fatigue, renal function, muscle damage and inflammatory activity, and cardiac damage following the two races. Our hypotheses were the following: (1) body weight loss and sodium depletion would be greater following the shorter race; (2) lower-limb and respiratory fatigue would be greater after the longer race; (3) renal alteration, muscle damage and inflammatory response may be greater following the longer race; (4) a higher release of cardiac troponin would be observed after the shorter race.

## 2. Material and methods

### 2.1 Experimental design

Two different races were studied (Costa Blanca Trails, November 2018; and Penyagolosa Trails, April 2019). Costa Blanca Trails race consisted of a 65 km circular route with a total positive elevation of 4200 m. Penyagolosa Trails race consisted of a 107.4 km non-circular route, starting at an altitude of 40 m and finishing at 1,280 m above the sea level, with a total positive and negative elevation of 5604 and 4356 m respectively. In line with the literature [2], participants in each of these two races will be henceforth referred as long trail (LT) and ultra trail (UT) runners, respectively. Measurements were performed with the same equipment, following the same procedures and by the same group of researchers. The day before the race, body composition, respiratory and lower limb strength assessments were conducted and a blood sample was collected. After the race, body weight (BW), respiratory and lower limb strength assessments were repeated and a second blood sample was collected. Lastly, a third blood sample was collected 24-h following the race. In both races, finishing times were obtained from race results and mean flat-equivalent running speed was calculated according to the procedure established by Saugy et al. [5].

### 2.2. Participants

All participants from both races received an invitation email to participate in the study. Those individuals who accepted the invitation were contacted by telephone and fully informed about the study procedure and inclusion criteria. Nineteen athletes (15 males and 4 females) and forty three athletes (26 males and 17 females) were selected from the LT and the UT races respectively to participate in the study, according to the following inclusion criteria: having previously completed at least one ultramarathon (>42 km); being free from cardiac or renal disease and from taking any medication on a regular basis. All subjects gave their written consent to participate and were also allowed to withdraw from the study at will. The same questionnaire was used to collect demographic and medical information as well competition history and training data from the three months prior to the race for both samples. The investigation was conducted in accordance with the Declaration of Helsinki and approval for the project was obtained from the research Ethics Committee of the University Jaume I of Castellon (Expedient Number CD/007/2019). This study is enrolled in the ClinicalTrails.gov database, with the code number NCT03990259 (www.clinicaltrials.gov).

### 2.3. Body composition

Body Mass Index (BMI) and percentage of fat mass (%FM) were evaluated using a bioelectrical impedance weight scale (Tanita BC-780MA, Tanita Corp., Tokyo, Japan). Measurements were performed in a fasted state with minimal clothing (running shorts and t-shirt), following the manufacturer’s guidelines. The skin and the electrodes were cleaned and dried before testing. Additionally, as in previous studies [24, 25], we also measured pre-race and immediately post-race body weight (BW) with the runner clothed in running wear and shoes (other items such as waist packs and hydration vests were removed and nothing was permitted in the runner’s hands).

### 2.4. Respiratory assessment

Pulmonary function testing was conducted using a portable desktop spirometer (Pony FX, Cosmed, Rome, Italy), with the participant seated and wearing a nose-clamp, in line with the American Thoracic Society and European Respiratory Society guidelines for spirometry standardization [26]. Measurements were performed the day before the race and within 15 min after the race by the same experienced investigator to ensure that maneuvers were carried out properly [27]. Forced vital capacity (FVC), forced expiratory volume in 1 s (FEV_1_), FEV_1_/FVC ratio and peak expiratory flow (PEF) were determined from the maximal flow volume loop (MFVL). Each participant performed three acceptable MFVL maneuvers lasting 6 s or longer each one. The spirometric maneuver with the highest sum of FVC and FEV_1_ was accepted. Furthermore, maximal inspiratory pressure (MIP) was measured to assess volitional maximal inspiratory strength using a handheld electronic device (Powerbrathe K5, HaB International Ltd, UK). This instrument has shown to be reliable [28]. Each participant performed three attempts and the best result was considered for analysis.

### 2.5. Lower limb strength assessment

Lower limb strength was evaluated using a squat jump (SJ) test. Participants were asked to jump as high as possible from a starting position with hips and knees flexed 80 degrees and hands stabilized on hips to avoid arm-swing. Jump height was estimated by the flight time measured with a contact platform (Chronojump, Barcelona, Spain) [29]. The test was performed twice, with a 90-seconds rest period between attempts [30]. Each individual’s best performance was retained for statistical analysis.

### 2.6. Blood sampling and analysis

Blood samples were collected from an antecubital vein by venipuncture using BD Vacutainer PST II tubes. Samples were centrifuged at 3500 rpm for ten minutes and kept at 4°C during transport to Vithas Rey Don Jaime Hospital (Castellon), where they were processed using the modular platform Roche / Hitachi clinical chemistry analyzer Cobas c311 (Roche Diagnostics, Penzberg, Germany), as previously published [13, 31, 32]. Biochemical results obtained immediately post-race were adjusted by employing the Dill and Costill method [33], using hematocrit and hemoglobin to determine the magnitude of plasma volume changes after the race in each participant [33, 34]. The following blood variables were considered for analysis: lactate dehydrogenase (LDH), creatine kinase (CK), sodium [Na+], C-reactive protein (CRP), Creatinine (Cr) and high sensitivity cardiac troponin (Hs-TNT). Moderate hyponatremia was defined as [Na+] lower than 129 mmol/L and mild hyponatremia as [Na+] between 129 and 134 mmol/L [16, 35]. Additionally, the Chronic Kidney Disease Epidemiology Collaboration (CKD-EPI) equation was used to calculate the glomerular filtration rate (GFR) [36]; and according to the Risk, Injury, Failure, Loss of function, and End-stage renal disease (RIFLE) classification [37], a GFR decrease greater than 25% from baseline was considered indicative of risk of Acute Kidney Injury (AKI). Hs-TNT was measured quantitatively with the new high-sensitive enzyme immunoassay based on electrochemiluminescence technology (ECLIA), using a Cobas e411 analyzer (Roche Diagnostics, Penzberg, Germany). The Limit of the Blank (LoB) of this assay is 3 ng/L and the Upper Reference Limit (URL), defined as the 99th percentile of a healthy population, 14 ng/L. The approximate hs-TnT equivalent to the upper limit of 30 ng/L for the 4th generation cardiac troponin T assay is 50 ng/L [38].

### 2.7. Statistical analysis

Statistical analyses were carried out using the Statistical Package for the Social Sciences software (IBM SPSS Statistics for Windows, version 22.0, IBM Corp., Armonk, NY). Normal distribution of the variables was verified through the Kolmogorov-Smirnov test (p<0.05). Blood variables and training and competition related data did not present a normal distribution, so non-parametric statistics were applied to assess these variables. Flat-equivalent running speed, pre-to-post change in BW and body composition variables (BMI and %FM) were compared between UT and LT runners using Student’s t-tests. Training and competition related data were compared between the two groups using Mann-Whitney U and Chi-Square tests.

A two-factor repeated-measures ANOVA was conducted, with ‘Fatigue’ (pre-race vs post-race) as within-factor and ‘Race’ (UT vs LT) as between-factor, to assess the evolution of respiratory variables (FVC, FEV_1_, FVC/FEV_1_ PEF, MIP) and SJ in the two groups. Friedman tests were conducted to appraise the evolution of blood variables (Cr, GFR, [Na+], CK, LDH, CRP and hs-TNT) from pre-race to 24 h post-race in each group (UT vs LT). Pairwise comparisons were performed using Bonferroni’s test (normally distributed variables) and Mann-Whitney U and Wilcoxon tests (non-normally distributed variables). Categorical data (natremic status, AKI risk and hs-TNT upper/below the URL) were analyzed by means of Chi-Square tests. The meaningfulness of the outcomes was estimated through the partial estimated effect size (η2 partial) for ANOVA and Cohen’s d effect size for pairwise comparisons. In the latter case, a Cohen’s D between 0.3–0.5 was considered small; between 0.5–0.8, moderate; and greater than 0.8, large [39]. The significance level was set at p-value <0.05 and data are presented as means and standard deviations (±SD).

## 3. Results

Thirty-two athletes (19 males and 13 females) completed the UT race with an average finish time of 21 h 23 min ± 3 h 28 min (167 ± 29% of winning time), while seventeen athletes (13 males and 4 females) completed the LT race with an average finish time of 11 h 1 min ± 1 h 41 min (146 ± 21% of winning time). The finishers/starters ratio in our sample was 74.4% and 89% for the UT and LT races. Considering all race participants, the finishers/starters ratios were 73.8% and 92.5% respectively, while the average finish times were 20 h 24 min ± 3 h 11 min and 11 h 29 min ± 1 h 55 min. Mean flat-equivalent running speed was significantly higher in LT compared to UT runners (9.92 ± 1.48 vs 5.75 ± 1.04 km/h; p<0.01; *d* = 3.5). Change in BW was not significantly different between UT and LT groups (1.5 ± 2.1% vs 2.4 ± 1.6; p = 0.12; *d* = 0.5).

Body composition data, training and competition related data from finishers of UT and LT are compared in Table 1. No significant between-group differences were identified in body composition variables. On the other hand, UT runners appeared to possess greater training experience (p = 0.04; *d* = 0.7) and being finishers of more races longer than 100 km (p<0.01; *d* = 0.6). Regarding training-related data of the 3 months prior to the race, no differences were found either in training days and hours or in running volume and weekly positive elevation. However, the percentage of runners who performed at least one weekly strength-training was significantly higher among UT participants (p = 0.02).

**Table 1:** Characteristics of ultra trail and long trail participants (mean ± SD).

	UT (n = 32)	LT (n = 17)	UT vs. LT	
			p-value	Cohen’s D
**Age** (years)	41 ± 6	41 ± 7	0.85	0.1
**BMI** (kg/m^2^)	22.8 ± 2.0	23.6 ± 2.5	0.27	0.3
**FM** (%)	15.4 ± 4.9	14.9 ± 4.8	0.73	0.1
**Number of years running**	8 ± 3	6 ± 3	**0.04**	**0.7**
**Number of races >100 km**	2 ± 3	1 ± 2	**<0.01**	**0.6**
**Weekly training days**	5 ± 1	5 ± 1	0.51	0.1
**Weekly running volume** (km)	70 ± 22	58 ± 24	0.16	0.6
**Weekly positive elevation** (m)	1772 ± 691	1493 ± 708	0.36	0.4
**Weekly training hours**	10 ± 4	9 ± 5	0.45	0.1
**Strength training** (yes/no)	81% / 19%	47% / 53%	**0.02**	-

**Abbreviations:** BMI, Body Mass Index; FM, fat mass; Strength training (yes/no), percentage of participants who performed at least one weekly lower-limb strength training in the previous 3 months.

Values of expiratory pulmonary function, MIP and SJ at pre-race and post-race in UT and LT runners are presented in Table 2. Univariate contrast analysis from repeated measures ANOVA showed a significant effect for ‘Fatigue’ on FVC [F = 5.99; p = 0.02; η2 partial = 0.12], FEV_1_ [F = 8.61; p<0.01; η2 partial = 0.17], PEF [F = 7.04; p = 0.01; η2 partial = 0.14], MIP [F = 64.69; p<0.01; η2 partial = 0.60] and SJ [F = 31.70; p<0.01; η2 partial = 0.41]. On the other hand, ‘Race’ factor significantly affected MIP [F = 7.20; p = 0.01; η2 partial = 0.14], while the ’Fatigue x Race’ interaction affected FVC [F = 7.17; p = 0.01; η2 partial = 0.12], FEV_1_ [F = 9.89; p<0.01; η2 partial = 0.19], PEF [F = 8.157; p<0.01; η2 partial = 0.16], MIP [F = 4.77; p = 0.03; η2 partial = 0.10] and SJ [F = 7.40; p<0.01; η2 partial = 0.14]. Bonferroni adjusted pairwise comparisons showed that, among UT participants, all respiratory variables showed a significant decline following the race: FVC (p<0.01; d = 0.5), FEV1 (p<0.01; d = 0.7), FEV1/FVC ratio (p = 0.03; d = 0.6), PEF (p<0.01; d = 0.6) and MIP (p<0.01; d = 0.8). Conversely, no significant pre-to-post changes were identified among LT participants regarding respiratory variables, except for MIP (p<0.01; d = 0.5). In addition, at post-race, LT runners displayed significantly higher values of MIP, compared with UT runners. Similarly, the UT group, unlike the LT group, evidenced a significant reduction in SJ performance following the race (p<0.01; d = 1.4); moreover, at post-race, a significant between-groups difference in SJ emerged.

**Table 2:** Spirometric-derived data, maximal inspiratory pressure and squat jump at pre-race and post-race in ultra trail and long trail participants (mean ± SD).

		UT	LT	UT vs LT	
		*(n = 32)*	*(n = 17)*	p-value	Cohen’s D
**FVC** (l)	**PRE-RACE**	4.42 ± 0.98	4.19 ± 0.93	0.55	0.3
	**POST-RACE**	3.91 ± 1.01 **	4.21 ± 0.86	0.35	0.3
**FEV**_**1**_ (l)	**PRE-RACE**	3.45 ± 0.74	3.29 ± 0.78	0.51	0.2
	**POST-RACE**	2.88 ± 0.99 **	3.31 ± 0.73	0.14	0.5
**FEV**_**1**_**/FVC** (%)	**PRE-RACE**	78.52 ± 4.92	77.88 ± 7.77	0.74	0.1
	**POST-RACE**	72.45 ± 13.98 **	78.13 ± 9	0.15	0.5
**PEF** (l/min)	**PRE-RACE**	8.08 ± 2.22	7.39 ± 1.8	0.29	0.3
	**POST-RACE**	6.72 ± 2.56 **	7.44 ± 1.95	0.33	0.3
**MIP** (cm H_2_O)	**PRE-RACE**	100 ± 28	115 ± 24	0.06	0.6
	**POST-RACE**	80 ± 24 **	103 ± 22 **	**<0.01**	**1.0**
**SJ** (cm)	**PRE-RACE**	24.2 ± 4.1	24.4 ± 3.7	0.90	0.1
	**POST-RACE**	18.4 ± 4.2 **	22.3 ± 4.6	**<0.01**	**0.9**

**Abbreviations** FVC, forced vital capacity; FEV_1_, forced expiratory volume in 1 s; PEF, Peak expiratory flow; MIP, maximal inspiratory pressure; SJ, squat jump.

* Significantly different from pre-race (p<0.05);

** Significantly different from pre-race (p<0.01).

**Fig 1:**
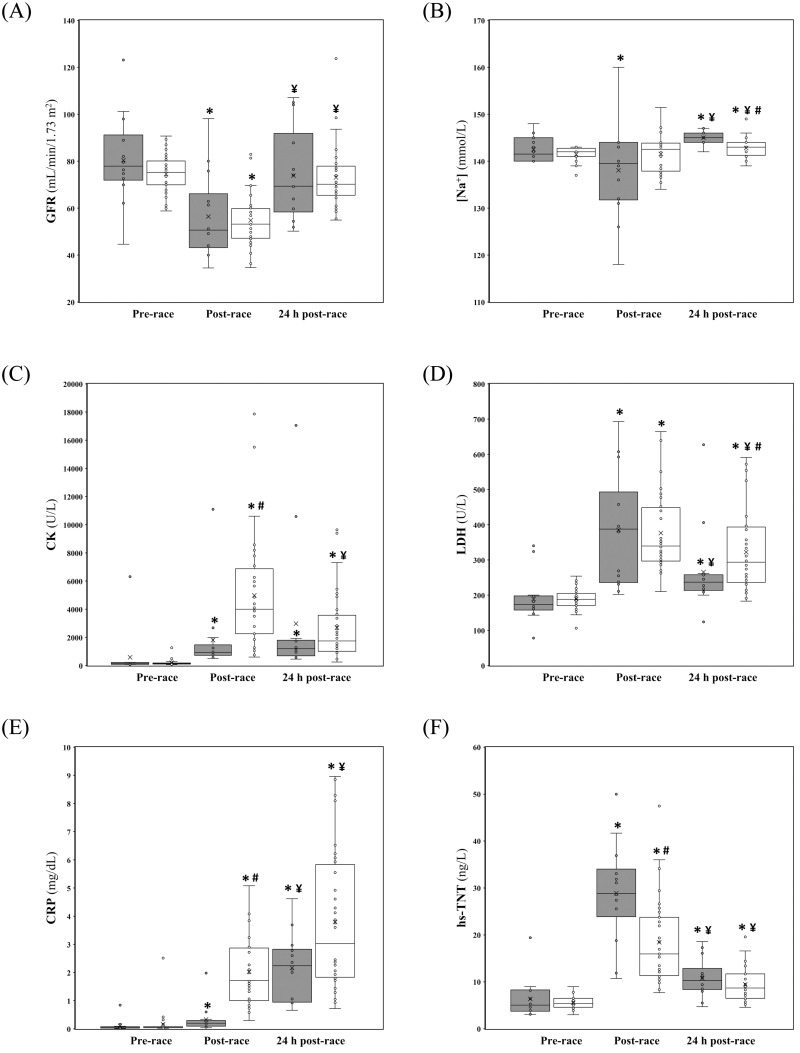
Glomerular filtration rate (panel A), sodium (panel B), creatine kinase (panel C), lactate dehydrogenase (panel D), C-reactive protein (panel E) and high sensitivity cardiac troponin (panel F) evolution from pre-race to 24-h post race in Long Trail runners (grey boxes) and Ultra Trail runners (white boxes). * Significantly different from pre-race (p<0.05). **#** Significantly different from Long Trail runners (p<0.05). ¥ Significantly different from the preceding time point (p<0.05).

Friedman analysis revealed significant differences in the evolution of Cr only among UT runners (χ^2(2)^ = 54.83, p<0.01). Subsequent Mann-Whitney U analyses revealed that Cr significantly increased at post-race (p<0.01, *d =* 1.5) and decreased at 24 h post-race (p<0.01, *d =* 1.2), but remained significantly elevated compared to pre-race (p = 0.01, *d =* 0.3). In addition, post-race Cr was significantly higher in UT runners compared with the LT group (p<0.01, *d =* 0.9). Significant differences were noted in the two groups in the evolution of GFR (UT: χ^2(2)^ = 49.00, p<0.01; LT: χ^2(2)^ = 12.00, p<0.01) (Fig 1A). Both groups showed a significant reduction in GFR following the race (p<0.01, *d =* 2.1; p = 0.01, *d =* 1.4; respectively) and a subsequent increase at 24 h post-race (p<0.01, *d =* 1.5; p<0.01, *d =* 0.9; respectively). No between-groups differences were identified in the percentage of runners who met the risk criteria for AKI at post-race (LT: 44%, UT: 56%; p = 0.42) and at 24-h post-race (LT: 7%, UT: 0%; p = 0.13). Significant differences were observed in the two groups in the evolution of [Na+] (UT: χ^2(2)^ = 10.43, p<0.01; LT: χ^2(2)^ = 10.43, p<0.01) (Fig 1B). At post-race, however, only LT runners showed significantly reduced values of [Na+] (p = 0.03, *d =* 0.6). At 24 h post-race in both groups [Na+] was significantly above pre-race levels (p<0.01, *d =* 0.8; p<0.01, *d =* 1.2; respectively). Moreover, [Na+] was significantly higher in LT runners as compared with UT group (p<0.01, *d =* 1.2) at that time point. No between-groups differences were identified in the distribution of normonatremic, mild hyponatremic and moderate hyponatremic cases at post-race (LT: 76%/12%/12%; UT: 97%/3%/0%; p = 0.42).

Significant differences were identified in the two groups in the evolution of CK (UT: χ^2(2)^ = 58.56, p<0.01; LT: χ^2(2)^ = 21.57, p<0.01) (Fig 1C). It significantly increased at post-race in both groups (p<0.01, *d =* 1.7; p<0.01, *d =* 0.6; respectively) and remained significantly elevated compared to pre-race (p<0.01, *d =* 1.5; p<0.01, *d =* 0.7; respectively). However, in UT runners, but not in LT runners, it significantly decreased from post-race to 24 h post-race (p<0.01, *d =* 0.7). Besides, post-race CK was significantly higher in UT runners as compared with LT group (p<0.01, *d =* 0.9). Significant differences were noted in the two groups in the evolution of LDH (UT: χ^2(2)^ = 58.56, p<0.01; LT: χ^2(2)^ = 22.43, p<0.01) (Fig 1D). In both groups, it significantly increased at post-race (p<0.01, *d =* 2.3; p<0.01, *d =* 1.6; respectively) and dropped at 24 h post-race (p<0.01, *d =* 0.5; p = 0.01, *d =* 0.9; respectively), but remained significantly elevated compared to pre-race (p<0.01, *d =* 1.6; p = 0.01, *d =* 0.8; respectively). At 24 h post-race LDH was significantly higher in UT runners compared with the LT group (p = 0.04, *d =* 0.50).

Significant differences were observed in the two groups in the evolution of CRP (UT: χ^2(2)^ = 58.56, p<0.01; LT: χ^2(2)^ = 28.00, p<0.01) (Fig 1E). In both groups, it significantly increased from pre-race to post race (p<0.01, *d =* 2.6; p<0.01, *d =* 0.6; respectively) and from post-race to 24 h post-race (p<0.01, *d =* 1.6; p<0.01, *d =* 2.1; respectively). Post-race CRP was significantly higher in UT runners as compared with LT group (p<0.01, *d =* 2.3). Lastly, significant differences were noted in the two groups in the evolution of hs-TNT (UT: χ^2(2)^ = 57.75, p<0.01; LT: χ^2(2)^ = 24.57, p<0.01) (Fig 1F). In both groups, it significantly increased at post-race (p<0.01, *d =* 1.0; p<0.01, *d =* 2.9; respectively) and dropped at 24 h post-race (p<0.01, *d =* 0.7; p<0.01, *d =* 2.3; respectively), but remained significantly elevated compared to pre-race (p<0.01, *d =* 0.9; p = 0.02, *d =* 1.1; respectively). Post-race hs-TNT was significantly higher in LT runners compared with the UT group (p<0.01, *d =* 0.3), although no between-groups differences were identified in the percentage of runners who surpassed the URL at post-race (LT: 77%, UT: 57%; p = 0.149) and at 24-h post-race (LT: 22%, UT: 13%; p = 0.44).

## 4. Discussion

The aim of this study was to assess the acute physiological effects of running a 107-km MUM, compared with a shorter 65-km MUM. Our first hypothesis was partially confirmed. No differences were observed regarding pre-to-post change in BW; but runners from the LT, unlike the UT group, evidenced a decrease in [Na+] following the race. Secondly, as hypothesized, UT runners showed greater post-race lower-limb and respiratory fatigue. Regarding biochemical variables, GFR returned to baseline values at 24 h post-race in both groups; muscle damage and inflammatory response was greater among UT runners, as predicted; and lastly, confirming our fourth hypothesis, acute release of hs-TNT was higher among LT runners.

Given the combination of substrate utilization and the liberation of glycogen-bound water during exercise, it has recently been argued that BW losses between 2–5% seem advisable to sustain body water balance and maintain euhydration during ultramarathons [40]. Our data was within this range for LT runners (2.41 ± 1.62%), however BW loss among the UT group was slightly lower (1.47 ± 2.14%). We hypothesized that BW loss would be greater following the shorter race as BW change has been positively correlated with running speed [24, 41, 42]. Our results showed that running speed was significantly higher in LT compared to UT runners, but the difference in BW change between groups did not reach statistical significance. On the other hand, only LT runners evidenced a decrease in [Na+] following the race. Indeed, 4 participants (23.5%) from LT race exhibited EAH (2 moderate hyponatremia and 2 mild hyponatremia), compared with only one case (3.1%) of mild hyponatremia among UT participants. None of those participants required medical attention and the incidence was within the rates previously reported in the field [41], even though one of the mild hyponatremic runners also presented a post-race CK indicative of rhabdomyolysis (11089 U/L). This latter result may support a previously suggested link between rhabdomyolysis and EAH [16, 43, 44].

Previous studies assessing respiratory fatigue following MUM have reported a significant reduction in expiratory pulmonary function irrespective of race length [8, 9]. Our results extend these previous studies to show that a LT (i.e. 65-km), unlike mountain races longer than 100 km, did not provoke significant fatigue in expiratory pulmonary function. However, MIP was significantly reduced following both races. These findings suggest that inspiratory muscle strength is more sensitive to fatigue subsequent to running a MUM, compared with expiratory pulmonary function [9, 45]. Hence, the addition of specific MIP training into the daily routine of these athletes might improve their performance [46, 47].

As noted in the introduction, it has been suggested that extremely long MUM (i.e., 330-km) provoke lesser lower-limb neuromuscular fatigue than shorter ones (i.e., 110–166 km) [5]. Yet, no previous studies, as far as we are aware, had compared strength loss following a LT compared to an UT. Our results show that SJ performance only decreased following the UT. Therefore, it seems that lower-limb strength loss increases correlative with MUM distance until it reaches a plateau [5, 48], and is lower in extremely long MUM probably as a consequence of a more conservative pacing strategy adopted by runners in such races [22, 23]. The absence of a pre-to-post change in SJ performance among LT runners contrasts, however, with a previous study conducted on a similar race (75 km with a total positive elevation of 3930 m) [6], where the authors reported a significant reduction of 20% in counter-movement jump (CMJ) height. Notwithstanding, differences in execution technique and muscle involvement between SJ and CMJ could explain such disagreement.

Concerning muscle damage, LDH release pattern was consistent following both races and coincides with previous studies in MUM [4, 18, 21]. It peaked immediately post-race and dropped at 24 h post-race, although its values remained significantly different from pre-race. The higher LDH concentration at 24 h post-race in UT runners, as compared with the LT group, also agrees with Rubio-Arias et al. [21] study. Regarding CK, Rubio-Arias et al. [21] found that post-race concentration was significantly higher following a 111-km MUM compared to a 54-km MUM, although runners from both races reached peak CK values immediately post-race and lowered these values at 24 h post-race. Our results concur showing higher CK values following the longer race; however, in our study the release pattern differs between UT and LT runners. CK peaked at post-race among UT runners and decreased at 24 h post-race, while LT runners showed an increase in CK from post-race to 24 h post-race. Previous studies on half and full road marathons have reported peak values of CK at 24-h post-race [31, 49]; while following longer MUM (i.e., 166-km), peak CK has been described to occur immediately post-race [4, 50]. In addition, during a flat 200-km race CK was reported to significantly increase from 19-fold pre-race value at mid race to 90-fold pre-race value at the end of the race [51]; similarly, a continuous rise in CK has been described during a 217-km MUM [52]. However, it seems that there is not an unanimous CK release pattern following endurance events longer than 42-km but shorter than 166-km. Differences between studies regarding samples’ training level and the ratio between distance and altitude change of the races could explain this disagreement. Regarding inflammatory response, both the results from Rubio-Arias et al. [21] and ours display a greater response following the longer race and an increase in CRP from post-race to 24 h post-race measurement. Notwithstanding, differences between UT and LT runners in CK and CRP in our study were significant only at post-race, whereas in the Rubio-Arias et al. [21] study they remained significant at 24-h post-race.

Muscle damage and inflammatory responses after running a MUM rarely results in adverse consequences among athletes; however, the release of excessive amounts of intramuscular proteins into the blood stream may negatively affect renal function, mainly in conditions of heat stress, dehydration, underlying renal problems, use of nonsteroidal anti-inflammatory drugs and inadequate training [19, 53]. Our results showed that in 56% of UT runners and 44% of LT the evolution of GFR from pre-to-post race met the risk criteria for AKI, without significant differences between races. Nevertheless, none of the participants in the study experienced an adverse event requiring medical attention during the race or within the first 24-h post-race. Moreover, no UT runner and only one LT runner met the criteria for AKI at 24-h post race. Similar results at post-race and 24-h post-race have been previously described following a multi-stage ultramarathon [17], a 104-km MUM [16], a 67-km MUM [18], a road marathon [32, 54] and a road half-marathon [55]. Therefore, it seems that no long-term renal function repercussions are expected following either road half & full marathons, or MUM.

Exercise-associated release of cardiac troponins has been postulated to be mainly related to exercise intensity (i.e., as opposed to exercise volume) [13, 56–59]. This fact could explain why post-race hs-TNT was higher among UT runners, as opposed to the results of muscle damage and inflammation biomarkers. Nevertheless, the difference in the percentage of runners (LT: 77%, UT: 57%) with post-race values of hs-TNT above the URL did not reach statistical significance and most of the runners in the two races displayed values below the URL at 24 h post-race (LT: 78%, UT: 87%). This rate of normalization (i.e., percentage of participants with hs-TNT values below the URL at 24 h post-race) is similar to the one previously reported following a road marathon (between 73 and 83%) [60–62] and a 91-km mountain bike race (82%) [58].

## 5. Conclusions

Our results suggest that expiratory pulmonary capacity and lower-limb strength are more fatigued following a longer MUM, while inspiratory strength loss is independent of distance. Acute muscle damage and inflammatory response appear to be greater following a longer MUM, although those differences disappear at 24-h post-race. Moreover, a different time-course of CK response was observed, in UT runners it peaked immediately post-race while in LT runners it did at 24-h post-race. On the other hand, the shorter race elicited a higher acute cardiac troponin release, yet the difference in the percentage of participants with values above the URL was not significant. Lastly, renal response seems not be affected by distance competition. None of the participants in the study required medical attention during the race or within the first 24-h post-race. Overall, our results provide a comprehensive view of how mountain ultramarathon distance competition may affect respiratory and lower-limb performance, cardiac, inflammatory, muscle damage and renal acute responses. On one hand, those outcomes could ease doctors with the interpretation of analytical values of ultratrail runners following a race. On the other hand, the knowledge of how muscle damage, respiratory capacity and lower-limb strength are affected by distance competition should be considered by coaches when planning their athletes’ training for those events.

The main limitation of the study was the comparison of two different sample sets for the two races. Notwithstanding, no between-groups differences were identified in age, body composition and endurance training data, so we consider that the results obtained are consistent enough to reach the abovementioned conclusions. Future studies should explore the effect of MUM distance in a same sample of athletes to corroborate our results.
